# Editorial: Women in nutritional epidemiology

**DOI:** 10.3389/fnut.2023.1147856

**Published:** 2023-02-03

**Authors:** Olga Castañer, Anna Tresserra-Rimbau, Letizia Bresciani, Rosa Casas

**Affiliations:** ^1^Centro de Investigación Biomédica en Red (CIBERESP) de Epidemiología y Salud Pública, Instituto de Salud Carlos III, Madrid, Spain; ^2^Cardiovascular Risk and Nutrition Research Group, Epidemiology and Public Health Program, Hospital del Mar Medical Research Institute (IMIM), Barcelona, Spain; ^3^Endocrinology Service, Hospital del Mar, Barcelona, Spain; ^4^Centro de Investigación Biomédica en Red (CIBEROBN) de Fisiopatología de la Obesidad y Nutrición, Instituto de Salud Carlos III, Madrid, Spain; ^5^Department of Nutrition, Food Sciences and Gastronomy, Xarxa d'Innovació Alimentària de Catalunya (XIA), School of Pharmacy and Food Sciences, University of Barcelona, Barcelona, Spain; ^6^Nutrition and Food Safety Research Institute (INSA), University of Barcelona, Coloma de Gramanet, Barcelona, Spain; ^7^Human Nutrition Unit, Department of Food and Drug, University of Parma, Parma, Italy; ^8^Department of Internal Medicine, Hospital Clinic, Institut d'Investigació Biomèdica August Pi i Sunyer (IDIBAPS), University of Barcelona, Barcelona, Spain

**Keywords:** women in science, sex differences, biomarkers, cancer, biomedicine, health outcomes, food sustainability

According to data from the UNESCO Institute for Statistics for 107 countries during 2015–2018, the participation of women represents only a third part of worldwide researchers (1). Gender balance is close to 50% in some regions, such as Central Asia, Latin America and the Caribbean, but this proportion is reduced to 30–35% in other regions, such as Europe and North America (2). Women are still critically underrepresented in scientific professions, and this underrepresentation is greater in some fields such as ICT (information and communications technology), and STEM (Science, Technology, Engineering, and Mathematics) research in particular (3). Throughout history, female researchers have faced a large number of systemic barriers (e.g., gender gap, pay gap, undervalued work or implicit bias) to advance in their global professional careers. Of notice, research women are less likely to be named as authors in the research team and their contributions are less acknowledged than their male counterparts (4). Women have usually received less scientific recognition. An example is Rosalind Franklin, a British scientist, whose contribution to the discovery of the structure of DNA was not recognized until long after her death. In the years since her death, she has won recognition among scientists for her research on the original Crick and Watson paper which had been denied by the scientific community (5). Recently, we can also find other examples such as the case of Jennifer Doudna and Emmanuelle Charpentier, who awarded the Nobel Prize in Chemistry in 2020 (6). They discovered one of gene technology's sharpest tools: the CRISPR/Cas9 genetic scissors. However, their contribution to this discovery had been relegated to a second position until then. It is essential to recognize the achievements of women in science. For that purpose, the aim of this Research Topic, *Women in nutritional epidemiology*, is to acknowledge achievements of women in both health and science fields related to nutritional epidemiology.

The issue collects 9 works presented by 9 excellent women researchers who work in the field of biomedicine and nutrition. The Research Topics include studies that associate food patterns or specific food components to obesity related factors, such as satiety, adiposity, and cardiometabolic factors. It also includes two articles in the context of hormonal-related cancers, such as ovarian and breast cancer, as well as a couple of studies investigating the association between biomarkers and health outcomes. Finally, two articles evaluated sex differences in nutritional studies and a test on the knowledge of food sustainability among youngsters.

Among the published articles of this Research Topic, two systematic reviews and meta-analysis were published. Liu et al. identified the current knowledge gaps on the impact of different heavy metals on breast cancer. Parilli-Moser et al. found moderate evidence on regular consumption of peanuts and the modulation of lipid metabolism, reducing triglyceride blood levels, without promoting weight gain. A randomized clinical trial led by Hernando-Redondo et al. provided high-quality evidence on both mid-and long-term satiety hormones, as a pertinent approach to weight loss on a weight loss intervention with a hypocaloric Mediterranean diet and physical activity promotion. Gong et al. presented the ovarian cancer follow-up study (OOPS) protocol, which is an on-going hospital-based large prospective longitudinal cohort study better understand the linkage between biospecimens and clinical data collected throughout the patient treatment, and reveal additional information about the prognosis of ovarian cancer. Moreover, three cross-sectional studies were published on this topic. The first one did not show conclusive results, since different levels of association between hemoglobin levels and preterm birth were observed (Elmugabil et al.). The second study, conducted by Laveriano-Santos et al., showed potential therapeutic effects of cocoa flavonoids against obesity, demonstrating an association between high cocoa consumption and lower risk of presenting abdominal obesity and better adiposity parameters. Finally, de Moraes Prata Gaspar et al. pointed out the importance to implement more sustainable practices within the university community. These conclusions were derived from results of 1,220 participants that completed the survey to evaluate the level of knowledge and perceptions of food sustainability in a university community from Spain. Additionally, a case-control study, which included 217 gestational diabetes mellitus (GDM) cases and 217 matched controls conducted by Li et al., suggested that the combinations of circulating fatty acids could be a significant marker of GDM development compared to individual fatty acids or their subgroups. Lastly, Garrabou et al. showed how sex influence is frequently underrated not only in biomedicine, also in nutritional and molecular medicine, leading to bias in scientific analyses.

Relevant scientific topics related to nutritional epidemiology leaded by women are highlighted in this Research Topic, which aims to encourage other women to continue contributing in this field, as well as in other scientific fields.

## Author contributions

OC and RC wrote the draft. All authors revised, discussed, and modified the text, agreed on the content, contributed to the article, and approved the submitted version.
